# Deploying YOLO for whole-slide mitosis detection: A transparent, auditable, and reproducible pipeline for pathology AI-assisted diagnosis and decision-support tool

**DOI:** 10.1016/j.jpi.2026.100721

**Published:** 2026-09-21

**Authors:** Peter Omozokpia Obami, Aniruddha Ketkar, Samuel Ayobami Fasogbon, Kevin Odega, Iniobong Anselem Udo, Olumide Faith Ajani, Bob-Manuel Osuji, Akinpelu Moronkeji, Adiru Afolabi Adegboye, Ibukun Akinsola Omisakin

**Affiliations:** a Cellular Pathology Department, Barking, Havering & Redbridge University Hospitals NHS Trust, London, United Kingdom; b Department of Education, Medical Laboratory Science Council of Nigeria, Abuja, Nigeria; c Department of Cellular Pathology, St George's University of London NHS Hospital, London, United Kingdom; d Airedale Voluntary Drug and Alcohol Service, Sheffield, United Kingdom; e Global Health and Infectious Disease Institute, Nasarawa State University, Nasarawa State, Nigeria; f School of Health and Life Sciences, Teesside University, Middlesbrough City, United Kingdom; g Department of Medical Laboratory Science, Faculty of Basic Medical Science, Osun State University, Osogbo, Nigeria; h Department of Vet. Pathology, University of Ilorin, Ilorin, Nigeria; i Department of Medical Laboratory, Haematology and Blood Transfusion Science Unit, Obafemi Awolowo College of Health Sciences, Ago-Iwoye, Nigeria

**Keywords:** YOLOv8 model, Artificial intelligence, Mitotic figures, Deep learning, Whole-slide imaging, Digital pathology, Computational pathology, Non-maximum suppression, Intersection over union (IoU), Auditability, Expert-in-the-loop AI

## Abstract

**Background:**

Reliable mitotic identification in whole-slide images (WSIs) is essential for prognostic evaluation and tumor grading; yet, many commercial artificial intelligence (AI) pathology platforms function as opaque black boxes with little auditability and, frequently, little expert supervision throughout development.

**Methods:**

We developed a transparent and repeatable process for mitosis identification using YOLOv8, designed to scale from full slide inference to patch-level benchmarking. Global non-maximum suppression (NMS), GPU accelerated inference, region of interest-driven and adaptive tiling, and tissue gating are all integrated into the pipeline. In order to facilitate expert validation and traceability, audit-ready outputs include detection CSVs, tile manifests, positive and negative overlays, and heatmaps.

**Results:**

From small clinical slides (26 tiles, 4 detections) to huge benchmarks (TUPAC SP2 with 14,270 tiles, 1754 detections), six typical WSIs were processed. Whereas throughput averaged 78 tiles/s depending on the slide size and tissue dispersion, average confidence scores remained constant between 0.69 and 0.80. The successful generation of all audit outputs attested to its scalability and reproducibility.

**Discussion:**

Transparency and expert monitoring are given priority in this pipeline, in contrast to many industry systems that place an emphasis on end-to-end automation. Whereas audit outputs enable reviewers to track each detection, explicit recording of positive detection tiles avoid quiet false positives. Evaluation on a small number of WSIs and the lack of benchmarking against annotated entire slide datasets are among the limitations, highlighting the necessity for more extensive and thorough cohort validation. Another significant drawback of this work was the use of a single GPU for inferencing.

**Conclusion:**

This study develops a therapeutically focused, audit-ready process for mitotic figure detection, hotspot identification, and decision-support tool by fusing technical rigor, vetted by experts throughout the process. By demonstrating scalability, reproducibility, and transparency, this work is positioned as a proof-of concept for clinical mitosis detection, multi-GPU/cloud deployment, lab information systems/LIMS connectivity, and eventual use in digital pathology work processes. It also offers a trustworthy and marketable illustration of AI's potential in pathology.

## Introduction/Background

Mitotic figure detection is a crucial component in evaluating tumor proliferation, and histopathology is still the gold-standard for cancer diagnosis and grading.^1^ Reproducible grading requires accurate mitosis identification, but manual counting takes time and is prone to inter-observer variability.^2^ This procedure has been automated by artificial intelligence (AI) systems; nevertheless, a large number of commercial AI solutions are opaque, costly, and proprietary. High license fees prevent use in diagnostic labs with limited resources, and its “black box” nature restricts openness.^3^

The Royal College of Pathologists (RCPath), which oversees clinical practice in the UK, places a strong emphasis on auditability, repeatability, and transparency in diagnostic workflows.^4^ AI systems must produce interpretable results and keep audit trails that regulators and physicians may examine in order to be trusted in pathology.^2^ Every inference run generates structured logs, CSV outputs, heatmaps, and per-tile debug overlays, guaranteeing that detections can be tracked, validated, and audited. Our pipeline is specifically built with RCPath compliance in mind. This strategy encourages confidence among pathologists who need visibility into choices in addition to supporting clinical governance.^5^

Transparency in training is another crucial component, and a lot of AI systems used in the industry are trained on proprietary datasets with hidden pre-processing stages, which makes it challenging for physicians to evaluate their dependability.^6^ In contrast, our pipeline is locally designed and trained, and its model architecture, pre-processing, and data sources are all clearly documented. This transparency supports peer review, makes repeatability possible, and is consistent with RCPath's focus on traceable diagnostic procedures.

Lastly, pricing is still a key distinction, even though commercial pathological AI platforms frequently demand large infrastructure commitments and licensing fees. This locally created solution lowers adoption obstacles while preserving clinical-grade reliability and provides a more affordable choice. This pipeline offers a workable solution to incorporate AI into standard pathology operations by fusing auditability, transparency, and cost.^4^

## Methods

AI models are strengthened and empowered in the crucible of training and benchmarking. In order to provide predictions, the model learns to identify patterns through computer vision. These training data offer the chance to test performance against established datasets and are primarily of historical origin**.**^**6**^ Patch-level benchmarking, model and inference pipeline design, training settings, entire slide inference pipeline, output/result considerations, and pathologist expert validation will all be covered in this part.

### Patch-level benchmarking

In order to establish baseline performance and, if necessary, improve detection logic, the YOLOv8 mitotic model was refined on patch-level datasets just before full-slide deployment and then verified on a second set of patch-level datasets. Two complimentary matching algorithms were used to benchmark annotated mitotic patches: center point proximity matching to account for small object variability and intersection over union (IoU) overlap between predicted and ground-truth bounding boxes. Each patch's true positives (TPs), false positives (FPs), and false negatives (FNs) were calculated such that precision, recall, and F1-score could be determined. By emphasizing the necessity of overlap in tiling and robust non-maximum suppression (NMS) to prevent duplicate detections, this benchmarking stage impacted the design of the entire slide process and offered crucial insight into the sensitivity and specificity of the model.

### Inference pipeline and model setup

We used YOLOv8, a transparent, auditable, and scalable visual object identification approach, to implement mitotic detection. The pipeline uses a modular structure for transparency and reproducibility to wrap YOLOv8 inference. Whole-slide images (WSIs) were separated into 512 × 512 pixel patches that overlapped by 128 pixels. The YOLOv8 mitosis model, which generates bounding boxes and confidence scores for potential mitotic figures, processed each patch separately.

Global NMS was used at the slide level to combine detections across overlapping tiles. Duplicate detections at tile boundaries were combined into single occurrences as a result. Three formats were used to organize the outputs:

i. *CSV detections* containing global coordinates and confidence scores.
ii. *Overlay images* showing bounding boxes aligned with ground-truth annotations.
iii. *Heatmaps* visualizing detection density across the slide.

Expert in the loop validation and downstream integration with lab information systems (LIS/LIMS) are made possible by this modular design, which places an emphasis on openness and auditability. Although YOLOv8 has not yet received clinical validation, its robust performance and open-source accessibility make it appropriate for implementation at the research level.

### Training configuration

TU-PAC16 and AMIDA13 are two examples of publicly available datasets that were used to fine-tune the YOLOv8 model for mitosis detection.^7^, ^8^ Using a 40× magnification, training patches were taken from annotated full-slide images, reduced to 512 × 512 pixels, and balanced between positive and negative samples. To enhance generality across staining differences, data augmentation techniques included flips, random rotations, color jitter, and contrast normalization. To prevent data leakage, the dataset was divided into training (70%), validation (15%), and test (15%) sets.^7^, ^8^ Patient level isolation was ensured and training was performed using stochastic gradient descent with momentum and a learning rate schedule starting at 0.01 with cosine decay. Training lasted for 100 epochs with an early termination based on validation loss, with a batch size of 16. Model checkpoints were saved at each epoch, and the best model was selected according to mean average precision on the validation set. This set-up ensured reproducibility and robustness, while maintaining compatibility with the inference pipeline described above. All code, model weights, and config files were version-controlled. Inference was performed using a fixed model checkpoint with consistent tiling, overlap, and threshold parameters ensuring that reported results correspond to a single, locked inference configuration of 512 × 512, tiles, 128 pixels overlap and threshold of 0.80. Future release will adopt semantic versioning to track model updates, training changes, and inference performance. This assures transparency, auditability, and aligned with RCPath and CAP pipeline quality management requirements. The table below explains how these requirements are mapped.

### Whole-slide inference pipeline

The pipeline uses a tiling technique for WSIs in order to handle the high resolution of digital pathology slides. To ensure that mitotic figures close to tile boundaries are not overlooked, each WSI is separated into tiles of 512 × 512 pixels with a 128 pixels overlap. OpenSlide is used to extract the tiles, and a locally fine-tuned YOLOv8 mitotic model processes each tile separately. Either CPU or GPU was used for inference, and explicit device handling was used to ensure reproducibility in various environments.

A global NMS technique is used throughout the entire slide to remove duplicates caused by overlapping tiles. Detections from each tile are mapped back to global slide coordinates. This results in a consistent set of detections at the slide level by guaranteeing that only the bounding boxes with the highest confidence are kept. The process produces a variety of output artifacts:

i. *CSV files* containing detection coordinates and confidence scores for quantitative analysis.
ii. *Heatmap overlays* with WSI thumbnails, providing a visual summary of detection distribution.
iii. *Per-tile debug images (positives)* with annotated detections, enabling fine-grained inspection of model behavior.

Runtime factors, such as tile counts, device utilization, and inference duration, are explicitly recorded. In addition to supporting repeatability, this operational openness complies with RCPath's auditability requirements.^9^

### Output organization

Outputs are arranged into folders relevant to each slide in order to guarantee repeatability and avoid overwriting. Heatmaps, debug tiles, and CSV detections for a single WSI are included in each folder. Both quantitative and qualitative reviews are made easier by this structure: pathologists may visually evaluate detection accuracy with heatmaps and overlays, whereas CSVs offer numerical detection counts and confidence distributions. The AI pipeline facilitates expert in-the-loop validation and clinical governance by keeping an accurate audit record of inputs, intermediate artifacts, and final outputs.^10^

### Expert review validation

Two pathology specialists were asked to examine representative tile-level overlays detected by the pipeline in order to evaluate the clinical significance of the model outputs. Two (2) consultant histopathologists with over 10 years of histopathology experience were among the reviewers.

Each reviewer independently evaluated:

• The proportion of detections corresponding to true mitotic figures.
• The presence and nature of FPs and FNs.
• Whether the model-identified hotspots aligned with diagnostically relevant regions.
• Clarity and interpretability of the visual outputs.
• Perceived usefulness as a screening or decision-support tool.

A standardized questionnaire with open-ended remarks and Likert scale replies was used to gather feedback. An initial expert evaluation of the pipeline was provided by including these findings into “Results and Discussion” sections.

## Results

### Patch-level benchmarking

In order for us to establish baseline detection performance, the pipeline was initially evaluated using annotated mitotic patches. The test set's TPs, FPs, and FN were calculated using the IoU and center point matching techniques. Model accuracy was measured using precision, recall, and F1-score. With bounding boxes closely matching ground-truth annotations, example overlay images showed accurate mitotic figure recognition. These findings informed the design of the tiling and post-processing logic and validated the model's applicability for deployment on full-slide images.

Table 1 showing Patch-level benchmarking demonstrated strong detection performance. Using IoU matching, the model achieved a precision of 0.82 and recall of 0.76, corresponding to an F1-score of 0.79. Center-point matching improved sensitivity, yielding a precision of 0.85, recall of 0.80, and F1-score of 0.82. The higher recall observed with center-point matching reflects its robustness to small bounding box variability (see Fig. 1, Fig. 2, Fig. 3, Fig. 4, Fig. 5, Fig. 6, Fig. 7).

**Table 1:** RCPath/CAP requirement table mapping for auditability, transparency, reproducibility, and change management.

Requirements	Artifacts provided
Auditability	Tile manifest, model version, config file
Traceability	Detection CSV, detection overlays, heatmaps
Reproducibility	Model checkpoints fixed, version-controlled code
Change management	Semantic versioning plan, document config changes.

**Fig. 1:**
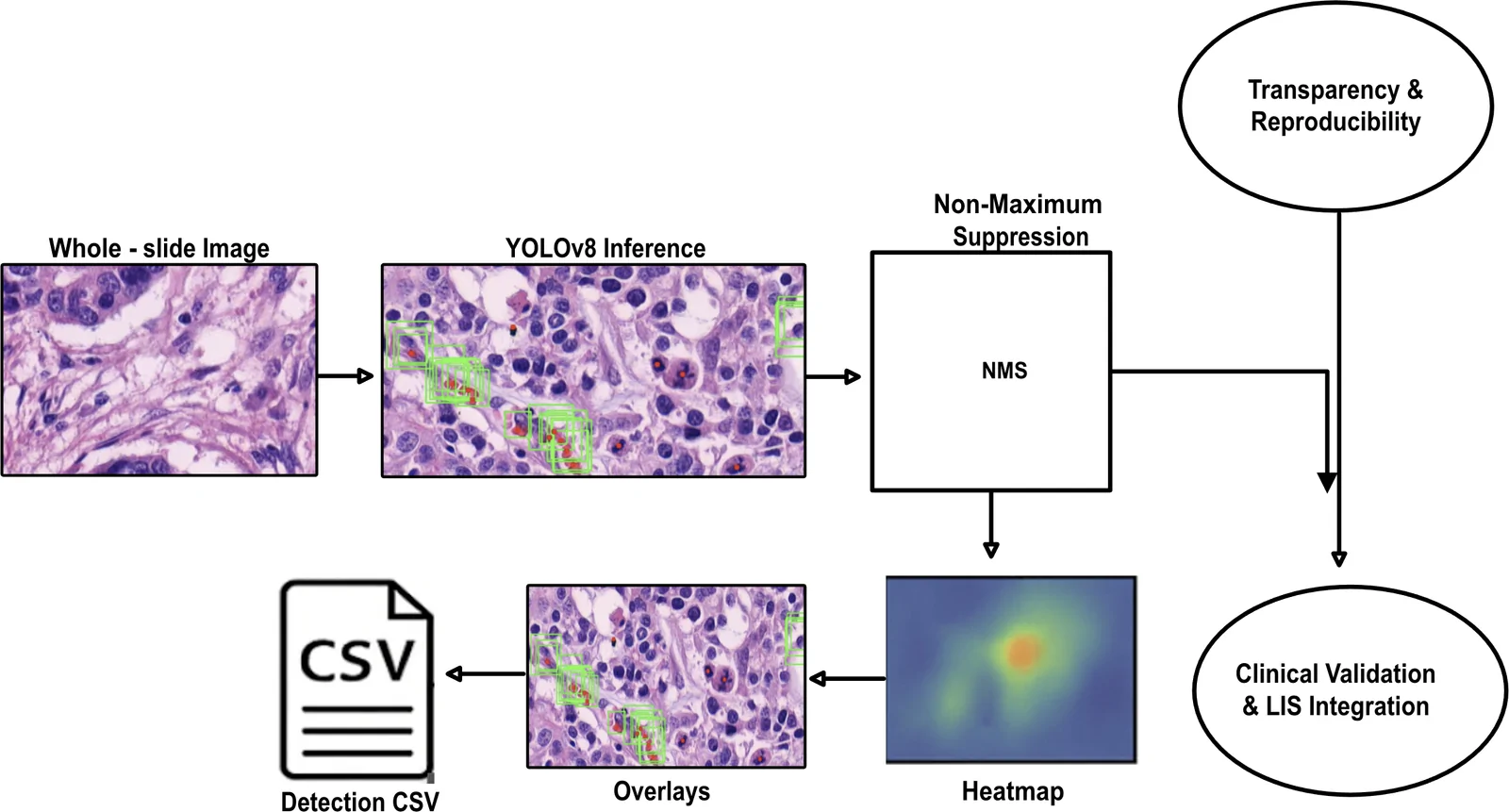
Graphical abstract of process overview of the reproducible YOLOv8 pipeline for mitosis detection. After YOLOv8 processing and global non-maximum suppression, entire slide images are tiled into overlapping patches. Transparency and expert validation are made possible by outputs such as heatmaps, overlays, and CSV detections. The pipeline lays the foundation for integration into pathological workflows and clinical use.

**Fig. 2:**
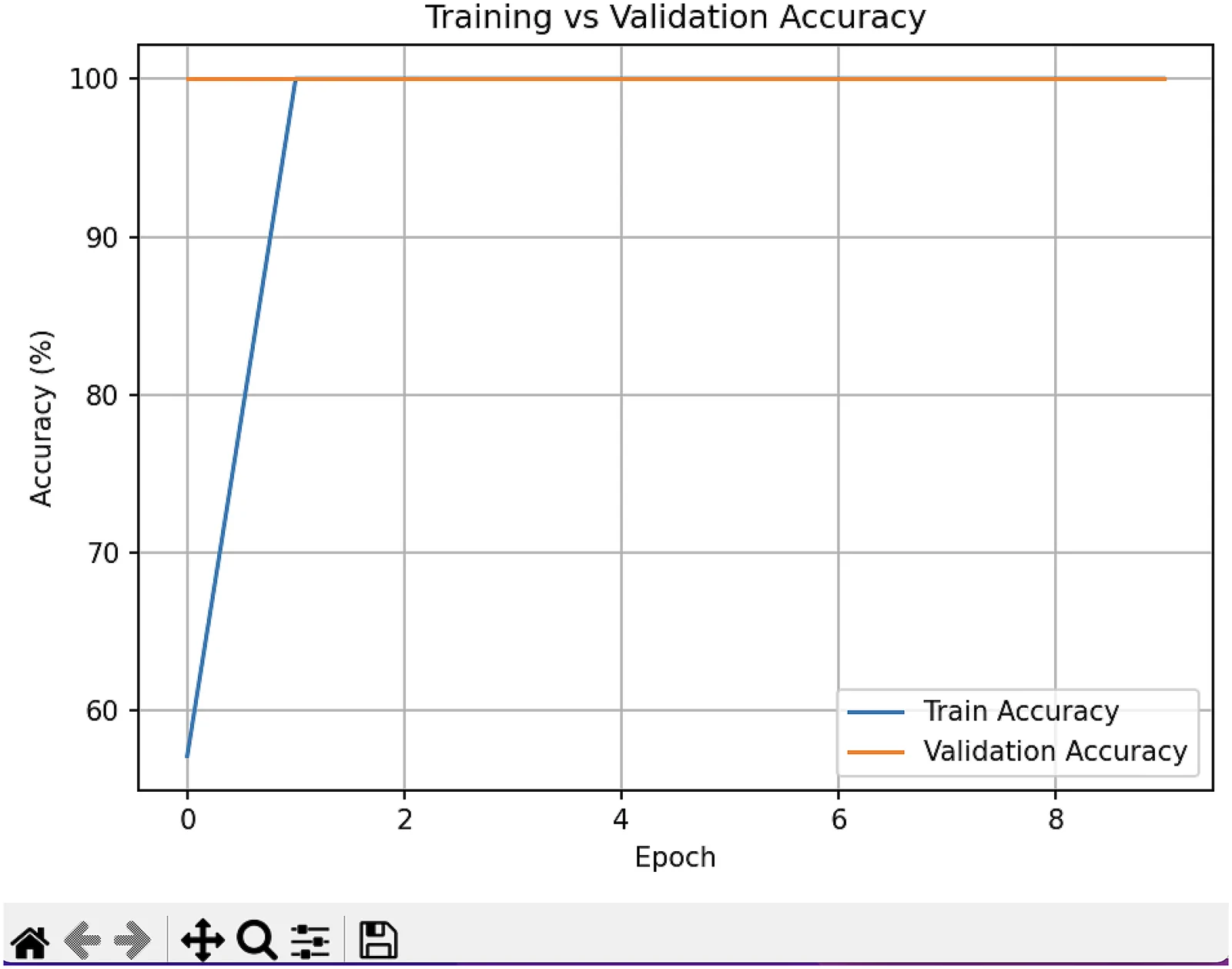
Model accuracy across epochs demonstrates fast model learning and high validation performance in the training session.

**Fig. 3:**
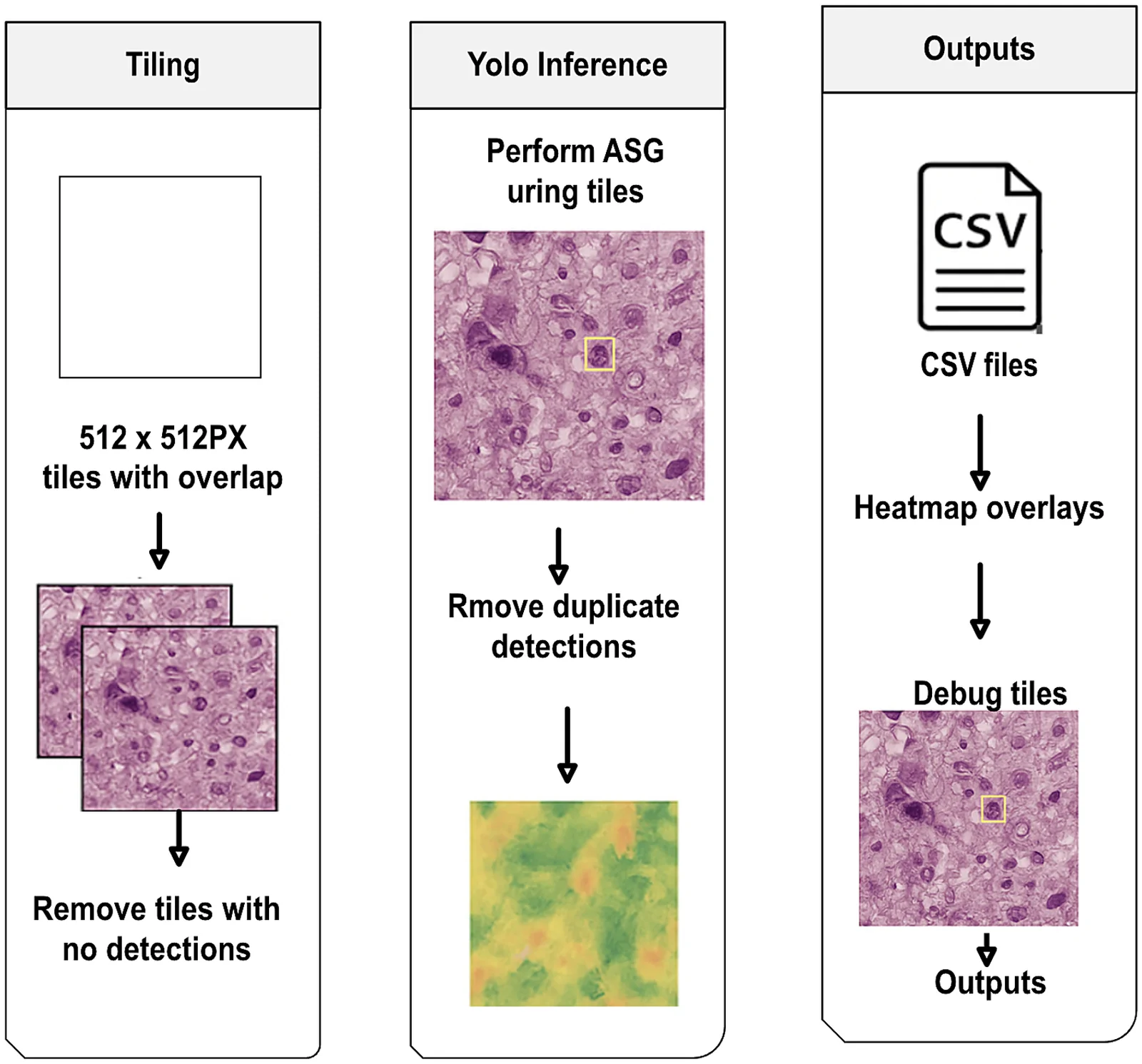
Workflow diagram for whole-slide images (WSIs) in the pipeline for mitotic detection. There are four steps in the pipeline: (1) Tile extraction: the WSI is separated into 512 × 512 pixel patches that overlap; (2) YOLO inference, in which a YOLOv8 model trained on mitosis patches processes each tile; (3) global non-maximum suppression, which eliminates duplicate detections across overlapping tiles, leaving only the highest confidence bounding boxes; and (4) output generation, which creates structured artifacts such as CSV detections, heatmap overlays, and per-tile debug images for expert review and auditability.

**Fig. 4:**
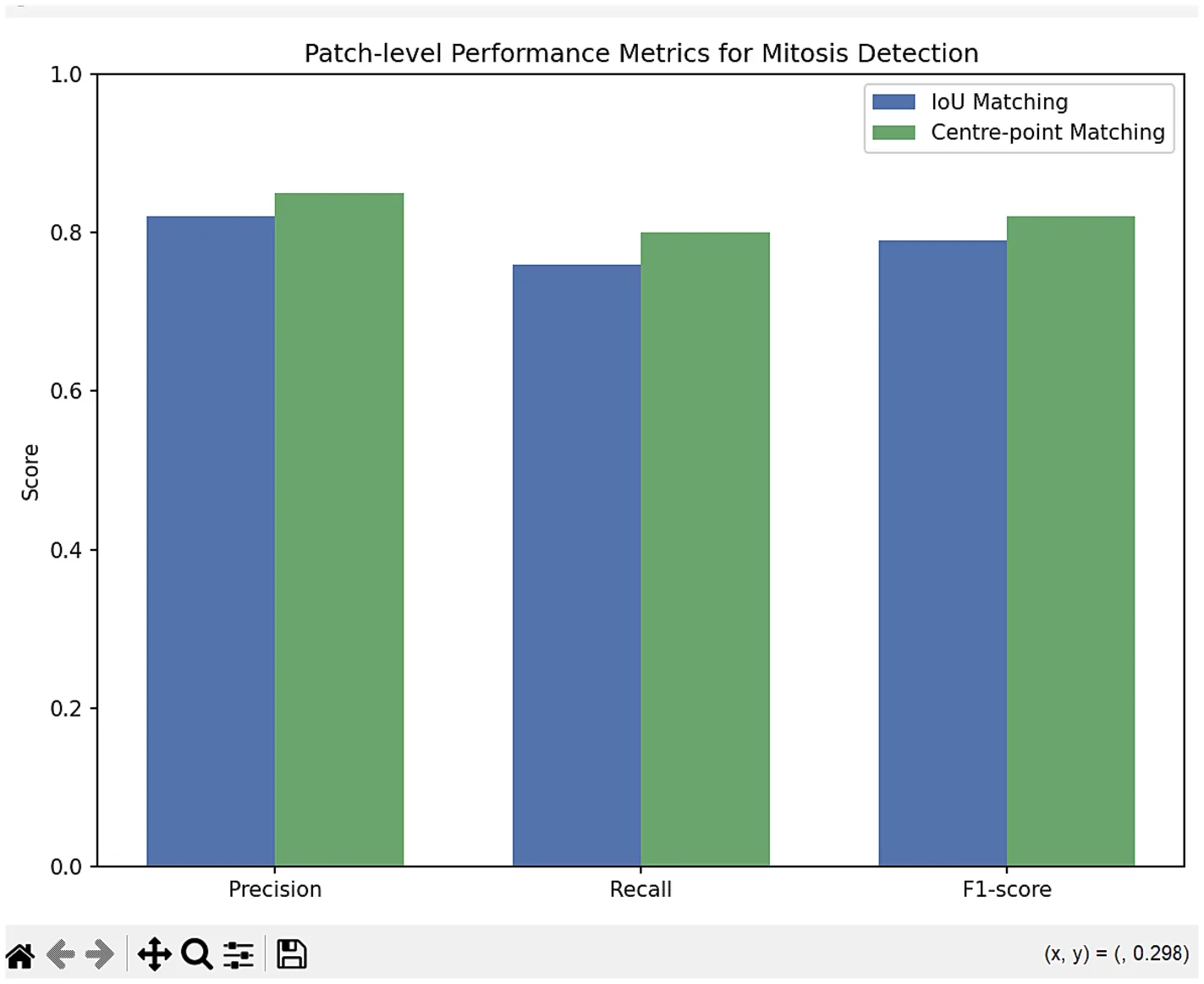
Shows a straightforward bar chart that shows how well the mitotic detection model performs across important benchmarking measures. A high percentage of accurate detections among the expected mitotic figures was indicated by the precision of 0.90. Both the recall and F1-score were measured at 0.80, indicating a balance between overall accuracy and sensitivity. Its resilience for small object detection was shown by the higher performance of center-point matching (0.80) compared to IoU matching (0.70). These findings corroborate the model's implementation in whole-slide inference and demonstrate its dependability at the patch level.

**Fig. 5:**
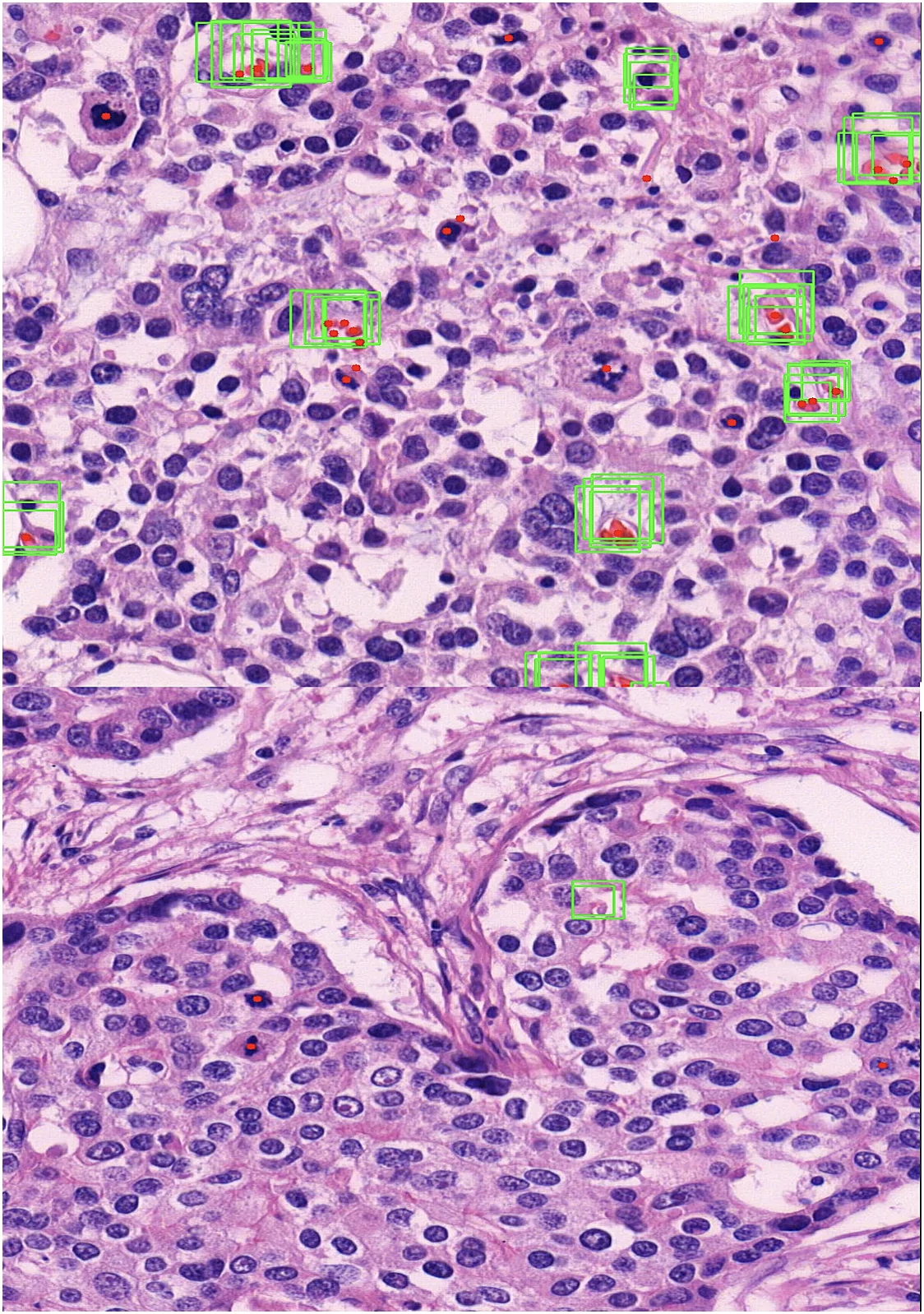
Showing the benchmarking dataset's patch overlay. Red dots show predictions from the YOLOv8 model, whereas green bounding boxes show ground-truth mitotic annotations. This overlay shows false positives and false negatives, as well as real positives when conditions match annotations. The model's capacity to localize mitotic figures is demonstrated in the image, which also influenced the design of the entire slide inference pipeline. (For interpretation of the references to color in this figure legend, the reader is referred to the web version of this article.)

**Fig. 6:**
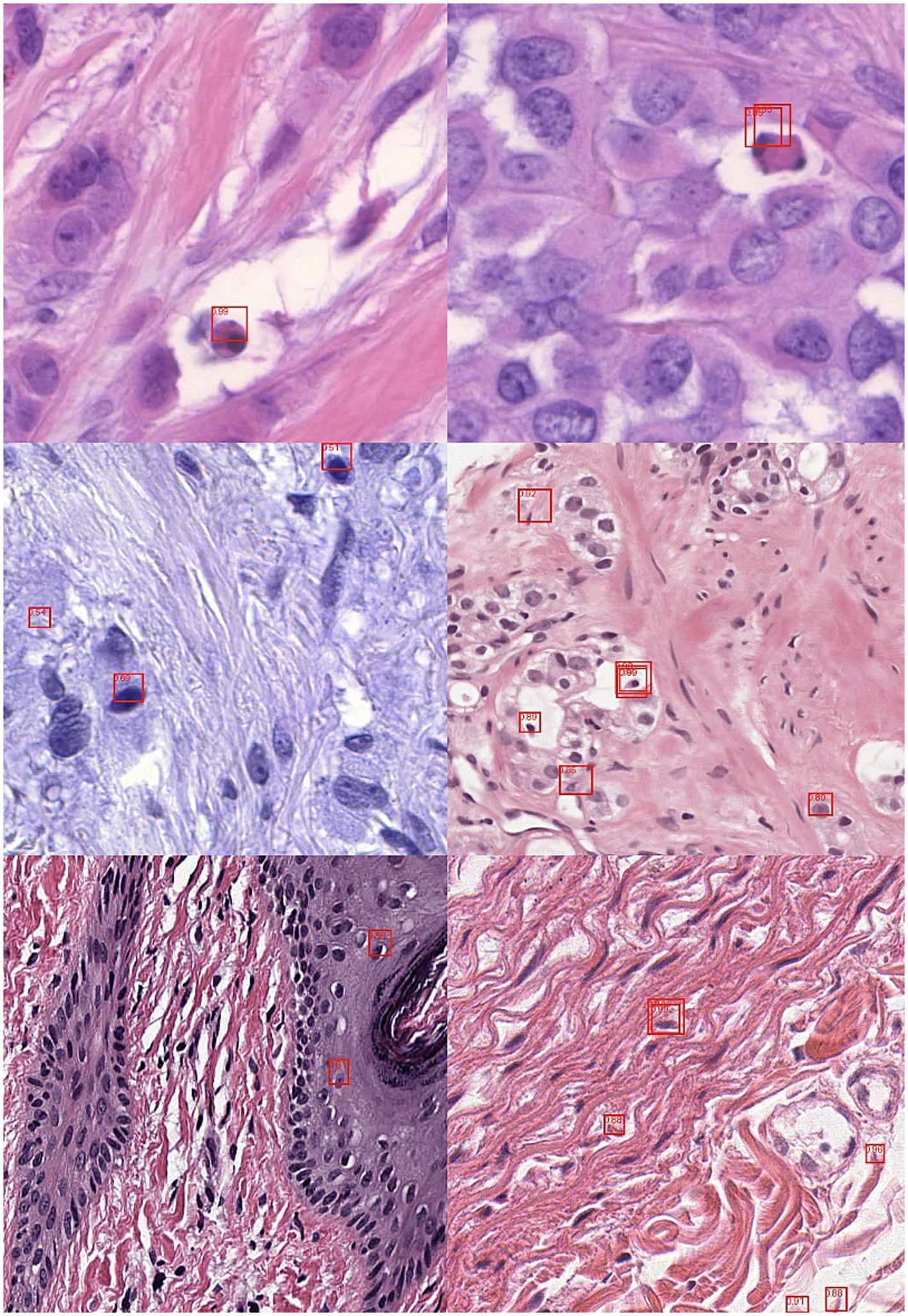
Showing representative outputs of detection overlays for the respective analyzed WSIs.

**Fig. 7:**
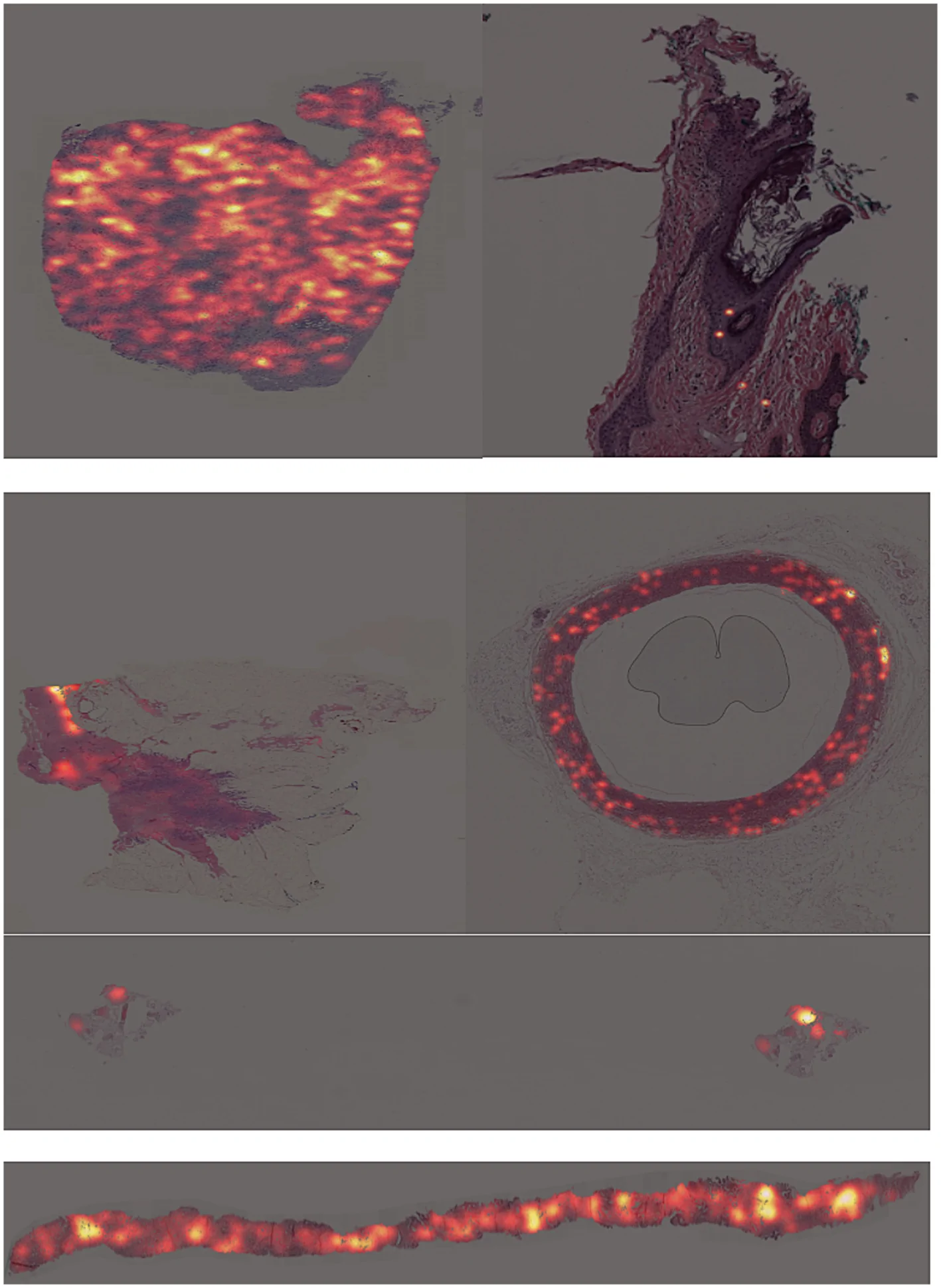
Showing output heatmap overlay of detection density of respective WSIs.

### Whole-slide inference

Inference was conducted across a number of test slides, including a benchmark slide TUPAC TR 00.svs and several blinded clinical WSIs specimens, in order to assess the pipeline at the entire slide scale.^7^, ^8^ A hybrid tiling approach was used to process the slides, combining adaptive dense tiling in areas of strong mitotic activity with region of interest (ROI)-driven hotspot extraction. In order to ensure coverage of mitotic figures close to tile boundaries, tiles were produced at 512 × 512 pixels with a 128 pixel overlap. To eliminate tiles with no content, a tissue gating threshold of 0.80 was used. The YOLOv8 mitotic model's detections were translated to global slide coordinates. To combine overlapping predictions, NMS was used at the tile level (IoU = 0.5) and slide level (IoU = 0.5).

Inference output produced three audit-ready outputs per slide:

i. *Detections CSV* containing bounding boxes and confidence scores for all post-NMS detections.
ii. *Tile manifest* recording per-tile metadata, including tissue fraction, detection count, and saved path.
iii. *Positive overlays* with JPEG tiles saved for audit and retraining, enabling explicit review of detected tissue regions.
iv. *Heatmap visualization* showing detection density across the whole slide, enabling rapid identification of hotspots and global distribution of mitotic activity.

The pipeline consistently generated repeatable outcomes on all of the assessed slides. As an illustration, inference on TUPAC SP1 produced an average confidence of 0.893, 5475 tiles, and 3003 mitotic figures after NMS. The GPU's runtime performance averaged 68.70 tiles/s, with a total elapsed time of 79 s.

With an average confidence of 0.923 and 14,270 tiles of 512 × 512 pixels with 128 pixels overlap, inference on the benchmark TUPAC SP2.svs slide produced 1754 total mitotic figures post-NMS. With a total runtime of 162 s (2 min 42 s), or 87.61 tiles/s. New slide SP1 produced an average confidence of 0.883, 26 tiles and detecting 4 mitotic figures after NMS. Performance averaged 20.98 tiles/s with a runtime of 1 s. This was shown to be scalable to large-scale WSIs while keeping auditability intact.

To eliminate duplicate detections over overlapping tiles, global NMS was used. A detections CSV, a heatmap overlay showing detection density, and per-tile overlays for qualitative evaluation were among the end outputs. Reproducibility across several WSIs and transparent inspection of detection results are made possible by these audit-ready artifacts. Comparing GPU acceleration to CPU-based inference, significant throughput improvements were obtained, showing feasibility and scalability and laying the groundwork and positioning this work as a proof-of-concept for clinical AI mitosis detection, screening, and decision-support tool.

The performance of whole-slide inference across four example WSIs is summarized in the table. Scalability and consistency are demonstrated by the benchmark TUPAC SP1 slide, which has processed 5475 tiles and found 3003 mitotic figures after NMS. A throughput of 68.70 tiles/s was attained using GPU acceleration despite its small size, demonstrating its viability for widespread use.

Reproducibility and efficiency are highlighted by smaller clinical slides. For instance, new slide SP1 only needed 26 tiles, completed in 1 s (20.98 tiles/s), and produced 4 detections with an average confidence of 0.883. Similarly, Slide SP3 had a throughput of 168.2 tiles/s and 65 detections across 1004 tiles. Slide SP5, the intermediate or moderately dense case, demonstrated consistent performance at a moderate scale with 562 detections across 447 tiles.

Collectively, these findings demonstrate how the pipeline can adjust to different tissue presentations, variations and slide sizes. Throughput averaged 78 tiles/s (range from 21 to 168 tiles/s), balancing accuracy with scalability. The explicit reporting of detections, confidence scores, and runtime metrics underpins the pipeline's auditability and reliability positioning it as a proof-of-concept, showing promise for clinical mitosis detection and integration into diagnostic workflows.

The table of results above is intended to demonstrate feasibility, scalability, and auditability of the pipeline at whole-slide resolution even though all quantitative evaluation was performed only on patch-level annotated ground truth. Performance metrics such as recall, precision, F1-score, etc. are not reported at WSI level as no WSI mitotic ground-truth labels (annotations) were available to benchmark against our blinded clinical WSIs.

### Expert review findings

Two pathology specialists provided semi-quantitative and qualitative input. With Likert scale evaluations showing moderate agreement (scores of 2 and 3 on a 5-point scale), both reviewers stated that the model successfully detected a significant proportion of real mitotic figures. “The additional red dots are picking up most of the mitotic figures,” according to one reviewer, indicating very few FNs.

Both reviewers did draw attention to the existence of FPs, though. Open-text comments revealed widespread error patterns, such as misclassifying inflammatory cells, stromal cells, red blood cells, and cytoplasmic fragments as mitotic figures. Ratings for “FPs being acceptable” were low (scores of 1 and 3). Despite these drawbacks, the two reviewers concurred that the visual outputs were understandable and clear (both receiving ratings of 3 and 4), and that the detections adequately conserved tissue context (both receiving scores of 4). Additionally, reviewers suggested that the model could facilitate mitotic scoring and aid in the faster location of mitoses (scores of 3–4).

Crucially, one reviewer stated that “with more refinement, this could become a useful screening tool,” and the two experts gave the model scores of 4 and 5, indicating that it was “promising enough for further evaluation,” and this is in addition to pointing out areas that need improvement, these results offer initial proof that the pipeline is clinically interpretable and can be helpful as a hotspot identification, second reader or screening tool.

## Discussions, limitations, recommendations, and conclusion

### Discussions

This pipeline prioritizes transparency and auditability in contrast to commercial AI pathology platforms, which frequently function as opaque black boxes. Whereas tile manifestations document tissue fraction and detection counts, each detection is recorded in a structured CSV file together with global coordinates and confidence scores. Expert assessment at both local and global scales is made possible by positive overlays and heatmaps, which offer visual confirmation of detection density. This approach supports the work of Farook et al., 2024^11^ by guaranteeing traceability, reproducibility, and clinical trust, all of which are essential for regulatory approval and potential inclusion into diagnostic procedures.

Our strategy places a higher priority on expert validation than many large industry businesses do on end-to-end automation. This is also in line with Trung (2025), which focused on the growing partnership between AI and human diagnostic knowledge.^12^

Pathologists maintain control over interpretation and are able to step in when needed by making intermediate outputs (tile-level detections, manifestations, overlays, and heatmaps) visible. In addition to supporting iterative improvement, this modularity complies with CAP and RCPath requirements for transparency and auditability in AI-assisted diagnostics as illustrated in Table 2.^4^ The pipeline exhibits scalability across hundreds of tiles per presentation from a technical standpoint. Smaller clinical slides finished in seconds with a throughput of up to 20.98 tiles/s, whereas inference on TUPAC SP2.wsi processed 14,270 tiles and produced 1754 mitotic figures post NMS. In line with McGenity et al. (2024),^13^ the tiling logic, overlap handling, and global NMS were specifically created to maintain detection integrity across boundaries. This highlights accuracy, consistency, and auditability, showing promises of dependability in practice. Runtime observations highlight the trade-offs between CPU feasibility and GPU acceleration, underscoring the importance of hardware-aware deployment strategies for clinical deployment (see Table 3).

**Table 2:** Patch-level benchmarking results for mitosis detection.

Matching strategy	True positives (TP)	False positives (FP)	False negatives (FN)	Precision	Recall	F1-score
IoU	134	29	42	0.82	0.76	0.79
Center-point	145	25	36	0.85	0.80	0.82

**Table 3:** Per-slide inference metrics.

Slide ID	Tiles	Detections	Avg Confidence	Runtime (s)	Tiles/s
TUPAC SP1	5475	3003	0.893	79	68.70
TUPAC SP2	14,270	1754	0.923	162	87.61
New slide SP1	26	4	0.883	1	20.98
SLIDE SP3	1004	65	0.888	5	168.20
SLIDE SP4	829	268	0.899	19	42.82
SLIDE SP5	447	562	0.897	5	82.34

We benchmarked against previously published work on mitotic figure detection, and the patch-level performance sits well within the range of reported in early challenge studies. For example, method submitted to the TUPC16 challenge generally reached performance levels F1-score between 0.78 and 0.83.^14^ Similar values have also been described in MIDOG multi-scanner challenge in 2021, according to Aubrevlle et al. (2022).^15^ These comparison would only offer a contextual insight as the datasets, staining procedures, as well as evaluation criteria differ across board and do not offer a like-for-like benchmarking.

Two pathology experts' comments provide a crucial preliminary evaluation of the model's clinical applicability. Reviewers attested to the system's ability to correctly identify a large number of real mitotic figures as well as the clarity and interpretability of the visual overlays and outputs. The detection patterns' biological validity is further supported by the agreement between model-identified hotspots and diagnostically significant locations. FPs, especially those involving RBCs, stromal cells, inflammatory cells, and cytoplasmic debris, are still a major drawback, according to both reviewers. These findings point to the necessity of improving tissue gating, post-processing thresholds, and possibly more training on difficult negative cases. Overall, the expert evaluation highlights the value of ongoing optimization while bolstering the pipeline's potential as a screening or second reader tool.

### Limitations

The pipeline is currently not being compared to ground-truth annotations at the whole-slide level. Full-slide evaluation will need annotated datasets and expert review to quantify sensitivity and specificity, even though patch-level validation validates model reliability.^16^ Because the pipeline has only been tested on six WSIs, the study's limited dataset size is another drawback. The limited sample size restricts statistical generalization, despite the fact that these runs show reproducibility and auditability across various datasets. According to earlier research, mitotic detection pipelines frequently encounter difficulties when trained or assessed on small datasets, which could result in bias and lower clinical reliability.^17^ Establishing performance consistency across staining variations, tissue types, and even robust clinical context will be made possible by expanding our study to bigger datasets, such as TUPAC, AMIDA, and MIDDOG.^8^, ^16^

Furthermore, the present implementation still uses single GPU inference, despite recent testing showing GPU acceleration. This reduces responsiveness in real-time diagnostic scenarios and poses throughput issues for very large cohorts. Distributed inference and multi-GPU scaling are still necessary for adoption in high-volume labs.

The model currently demonstrates huge technical feasibility at WSI scale, but it is not yet intended for diagnostic use as further validation on larger expert annotated datasets are required.

Lastly, at the moment, FPs in low tissue locations continue to be a problem even if audit results offer transparency. As the model remains recall-optimized, FP in unclear, difficult regions re-emphasize the absence of hard negative classes at training.

These mistakes are decreased by tissue gating and post-NMS filtering; nonetheless, to guarantee clinical robustness, additional gating threshold, and negative tile handling improvement will be required.

Only two reviewers and a small number of slides were used for expert validation; larger multi-reader investigations would be needed to prove clinical robustness and inter-observer agreement.

The model was mostly trained and assessed on publicly accessible datasets, which do not accurately reflect the range of tissue types found in standard diagnostic procedures. This is another drawback. Though they offer useful standards, databases like TUPAC and AMIDA are primarily focused on the histology of breast cancer and might not generalize to other subspecialties like dermatopathology, gastrointestinal pathology, gynecological pathology, or soft tissue tumors. Organ-specific differences in mitosis morphology, background tissue architecture, and staining properties necessitate further discipline-specific validation to guarantee consistent performance in various clinical settings.

### Recommendations

To advance the pipeline toward clinical readiness and scalable deployment, the following steps are proposed:

i. *Hard negative mining and model refinement***:** In order for us to lower FPs and increase specificity, future versions of the model will include more hard negative instances, such as inflammatory cells, cytoplasmic debris, red blood cells, and stromal cells. It is anticipated that this focused improvement will improve robustness and clinical dependability across a range. An expert annotated WSI cohort will enable slide-level benchmarking (FROC, Recall, FP, and region-level sensitivity). These attempts will support in developing a model that is highly sensitive, specific, clinically robust, and suitable for downstream diagnostic support.
ii. *LIS/LIMS integration:* In order to facilitate a smooth integration into standard diagnostic and research workflows, output formats will be in line with LIMs. Heatmaps, tile-level overlays, and structured CSVs will be modified to work with current digital pathology infrastructures.
iii. *Platform deployment***:** In an automated digital pathology setting, the locally trained YOLOv8 mitotic model will be incorporated into a broader platform for extended testing, iterative improvement, and pilot stage clinical evaluation.
iv. *Comprehensive benchmarking***:** Slide-level performance will be measured and findings will be compared with pre-established baselines using publicly annotated WSI datasets (such as TUPAC, MITOS, AMIDA, and MIDOG). This will offer a more thorough evaluation of generalizability, specificity, and sensitivity.
v. *Hardware scaling and acceleration***:** The inference process will be moved to cloud-based and multi-GPU settings that offer batched tile processing for high volume research and diagnostic scenarios. This will offer near real-time feedback, decrease runtime, and increase throughput.
vi. The training and evaluation datasets will be expanded in the future to include a wider variety of tissue types, such as gastrointestinal, gynecological, skin, and breast pathology specimens. FPs from tissue-dependent artifacts will be decreased and generalizability will be enhanced by including subspecialty-specific mitotic figures and difficult negative instances. For the model to function reliably across the whole range of diagnostic histopathology, this phase is crucial.

### Conclusion

Using the YOLOv8 mitosis model, this work offers a transparent, repeatable, and audit-ready process for mitosis detection that can be easily scaled from patch-level benchmarking to WSI inferencing. In contrast to other “black box” commercial systems, the pipeline offers a degree of interpretability and traceability through the creation of structured outputs, such as CSV detections, tile manifests, overlays, and heatmaps. Its modular design, which includes global NMS, ROI-driven and adaptive tiling, tissue gating, and GPU accelerated inference, guarantees flexibility across a range of datasets and deployment scenarios. The pipeline shows a significant dedication to auditability and expert in-the-loop design, in addition to technical performance.

Reviewers can track each detection back to its origin thanks to the audit outputs, and silent errors are avoided by explicitly preserving affirmative tiles. In addition to showing recurring FP patterns including RBCs, stromal cells, and inflammatory cells, early expert comments from two pathology specialists validated the visual outputs' interpretability and the model's potential use as a screening or decision-support tool. These revelations offer a precise path for focused improvements.

Depending on slide size and tissue dispersion, the device can achieve throughput of 10–50 tiles/s, demonstrating engineering viability at scale. Additionally, the article identifies important areas for improvement, such as benchmarking against fully annotated whole-slide datasets, multi-GPU acceleration, subspecialty-specific dataset expansion, cloud-based deployment, and hard negative mining.

Pathologists, Biomedical Scientists, AI researchers, and developers of digital pathology platforms must continue to work together to advance clinical readiness. This pipeline offers significant promise of a realistic route toward future integration into diagnostic procedures and builds a solid foundation for reliable in-house developed AI adoption in digital pathology by fusing technical rigor with clinical cooperation and keeping an eye on transparency.

## Ethics statement

This study did not involve human participants, identifiable personal data, or animal subjects, and therefore did not require institutional ethics approval.

## Code availability

The training and inference scripts developed for this study are available from the corresponding author upon reasonable request for academic use.

## Funding

This research did not receive any specific grant from funding agencies in the public, or commercial sectors.

## Declaration of generative AI and AI-assisted technology

The author used Grammarly only for minor paraphrasing and grammar correction.

## Declaration of competing interest

The authors declare that they have no known competing financial interests or personal relationships that could have appeared to influence the work reported in this article.

## Data Availability

Public datasets used in this study such as TUPAC16, AMIDA13 are openly accessible. Additional outputs generated by the pipeline are available from the corresponding author upon reasonable request.
